# Suicide after involuntary psychiatric care: a nationwide cohort study in Sweden

**DOI:** 10.1016/j.lanepe.2025.101504

**Published:** 2025-11-04

**Authors:** Leoni Grossmann, Fred Johansson, Seena Fazel, Ralf Kuja-Halkola, Björn Bråstad, David Mataix-Cols, Lorena Fernández de la Cruz, Bo Runeson, Paul Lichtenstein, Zheng Chang, Henrik Larsson, Isabell Brikell, Brian D'Onofrio, Ronnie Pingel, Christian Rück, John Wallert

**Affiliations:** a Centre for Psychiatry Research, Department of Clinical Neuroscience, Karolinska Institutet, Stockholm, Sweden, and Stockholm Health Care Services, Region Stockholm, Sweden; b Department of Psychiatry, Warneford Hospital, University of Oxford, Oxford, United Kingdom; c Department of Medical Epidemiology and Biostatistics, Karolinska Institutet, Stockholm, Sweden; d Department of Clinical Sciences, Lund University, Lund, Sweden; e School of Medical Sciences, Örebro University, Örebro, Sweden; f Department of Global Public Health and Primary Care, University of Bergen, Bergen, Norway; g Department of Biomedicine, Aarhus University, Aarhus, Denmark; h Department of Psychological and Brain Sciences, Indiana University, Bloomington, IN, USA; i Department of Statistics, Uppsala University, Uppsala, Sweden

**Keywords:** Suicide, Coercive psychiatric care, Compulsory admission

## Abstract

**Background:**

Little is known about the risk of suicide in individuals treated against their will in involuntary psychiatric care (IPC). This population-based study provides a first comprehensive description of suicide among individuals who experienced IPC.

**Methods:**

We studied all individuals discharged from IPC in Sweden from 2010 through 2020. Clinical and sociodemographic characteristics are reported followed by suicide risk for the complete IPC population and stratified by sex, age, IPC history, and diagnostic category. Crude and adjusted relative risks compared to all individuals discharged from psychiatric in- and outpatient care and the general population were estimated using Poisson regression. Suicide methods, seasonal trends, and geographical variance are also reported.

**Findings:**

We identified 72 275 patients treated in IPC with a total of 134 514 inpatient care episodes (mean age = 44·8 years, 37 462 [51·8%] males). Of these, 2104 (2·9%) died by suicide over a median follow-up time of 4·4 years (IQR: 1⋅8–7⋅5). Suicide decedents were younger, more often male, single, diagnosed with personality and substance use disorders, and had a history of self-harm and IPC, compared to those who did not die by suicide. The absolute risk (crude incidence rate (IR) per 100 000 person-years) for all IPC patients was highest closest to discharge (IR_1month_ = 2941 [2538, 3408]) and decreased thereafter (IR_5years_ = 738 [705, 773]). Suicide risk in IPC patients was elevated relative to psychiatric inpatients (crude IR ratio (IRR)_5years_ = 1·57 [1·48, 1·65]), psychiatric outpatients (IRR_5years_ = 3·77 [3·58, 3·97]), and the general population (IRR_5years_ = 55·52 [52·65, 58·54]).

**Interpretation:**

We found substantial risk differences in distinct subgroups of IPC patients and an excess suicide risk among IPC patients compared to other clinical populations. These findings warrant further investigation as they could inform clinicians and policy makers regarding potential risk stratification, monitoring, and care. Preventing suicides after IPC should be a priority.

**Funding:**

10.13039/501100004359VR, ALF Medicine, 10.13039/501100018713CIMED, 10.13039/501100006636FORTE, and Söderström König Foundation.

**Research in context**

**Evidence before this study**

We conducted a systematic search in PubMed, Web of Science, PsycINFO, CINAHL, Cochrane Central Register of Controlled Trials, and Embase for original research articles that were published between January 1, 2000 and December 1, 2024. The search strategy included key search terms such as (involuntar∗ OR coerc∗ OR compulsor∗) AND (psychiatr∗ OR mental∗) AND (admission∗ OR commit∗ OR treatment∗ OR hospital∗ OR stay∗ OR inpatient∗). Only a few studies have examined suicidality exclusively among involuntary psychiatric inpatients, despite this being one of the most acutely ill patient groups with a correspondingly elevated suicide risk. We found very little descriptive information on suicide following involuntary care. Also, due to the low base rate of suicide, suicidal ideation or suicide attempts have often been studied as a proxy for suicide death. However, the translation of such proxy findings for suicide death is limited given that only one in twenty suicide attempts results in suicide death. Furthermore, most identified articles suffered methodologically from selection bias, small samples sizes, and a lack of comparison groups. Regarding the risk of suicide, most studies report an increased suicide risk after involuntary psychiatric care, however, a few also conversely report a lower risk of suicide or a lack of any significant association between coercive care and suicide risk. A potential explanation for the contradictory evidence could be the use of different follow-up times across studies. Also, only investigating a single time-point and thereby assuming a constant suicide risk after discharge limits the interpretability of previous findings. Nonsignificant results might be due to low statistical power rather than true null associations. Taken together, the literature highlights a clear gap in understanding short- and long-term risk of suicide after involuntary psychiatric hospitalisation.

**Added value of this study**

To our knowledge, this is the largest cohort study and first comprehensive analysis of death by suicide and suicide risk in individuals who experienced involuntary psychiatric hospitalisation. We used high-quality, linked nationwide register data on an unselected, total population cohort of all patients admitted to involuntary psychiatric care in Sweden from 2010 through 2020 (>73 000 individual patients, >130 000 care episodes). This allowed us to calculate clinical population estimates for the risk of suicide across different follow-up times after involuntary care and also compare the risk to other psychiatric patients and the general population. We examined the suicide risk stratified by sex, age, and different diagnostic categories. Our findings highlight demographic and clinical subgroups of involuntary patients who are most affected by suicide. Individuals diagnosed with personality or substance use disorders had a higher suicide rate compared to those with schizophrenia or bipolar disorder. Overall, patients discharged from involuntary care had the highest suicide rate compared to all comparison groups. In addition, the excess risk in involuntary psychiatric patients compared to all psychiatric inpatients increased over longer follow-up times. Our estimates provide a first thorough analysis of both the absolute and relative suicide risk in involuntary psychiatric care patients to date.

**Implications of all the available evidence**

Quantitative information on the suicide risk after involuntary psychiatric care is essential to provide appropriate and timely suicide prevention for these patients. Our analysis shows that involuntary care patients with either substance use disorder or personality disorder have a higher suicide risk and emphasises high-risk periods following discharge from care. These findings lay the groundwork for improved risk stratification and tailored follow-up after involuntary care. Identifying individuals most at risk is an essential first step in directing resources and efforts for suicide prevention.

## Introduction

Suicide is a global public health concern accounting for 1·5% of all deaths worldwide.^1^ Nearly half of all who died by suicide had been in contact with psychiatric services three months prior to death^2^ and psychiatric disorders are robustly associated with high suicide risk.^3^, ^4^, ^5^, ^6^, ^7^, ^8^ Psychological autopsy studies further converge to indicate that clinical factors, such as the presence of mental disorders, show the strongest association with suicide.^9^

Involuntary psychiatric care (IPC) can be a lifesaving intervention for suicidal crisis in the context of a severe mental disorder and refusal of care.^10^ However, there is a general lack of information on the suicide risk after discharge from IPC, including a baseline description of patient characteristics when they leave the hospital after IPC. This is a remarkable knowledge gap, given that these patients are arguably more ill than voluntary psychiatric patients and demand substantial medical and legal resources. According to the Swedish Compulsory Psychiatric Care Act (1991:1128), involuntary care may only be given if the patient simultaneously suffers from a severe mental disorder, has an indispensable need for psychiatric care, and refuses care or is not able to provide consent. Overall, these criteria are comparable to those in other European countries. Unique to only a few European countries including Sweden, the risk of harm to self or others is only a consideration, not an explicitly required criterion.^11^ Involuntary care practices are not only influenced by legislation but also clinical practice and social contexts.^12^ Understanding different aspects of IPC and suicidality is essential for clinicians to effectively assess suicide risk and allocate appropriate resources to follow-up care. Such knowledge is also crucial to facilitate evidence-based policy development. Research on suicidality among IPC patients after hospital discharge can further shed light on the mechanisms of acute suicide risk and holds potential for improved patient management, monitoring, and care.

Using nationwide register data with up to 11 years of follow-up, we conducted the first population-based study of suicide in IPC patients after discharge from hospital. We report (i) sociodemographic and clinical characteristics, (ii) total and stratified suicide risk, (iii) suicide risk during one month up to five years after hospital discharge, (iv) relative suicide risk for IPC patients compared to all psychiatric inpatients, all psychiatric outpatients, and the general population, and (v) suicide method, geographical and seasonal patterns of suicide.

## Methods

### Design and population

We conducted a population-based descriptive register study including all involuntary psychiatric care inpatients in Sweden from January 1st 2010 through December 31st 2020. No age restriction was applied for study inclusion. The start of an IPC episode was defined as the date of admission to inpatient psychiatric care or medical care under the Compulsory Psychiatric Care Act (1991:1128). The end of the episode was defined as the date of discharge from psychiatric inpatient care (also used as start of follow-up for risk analysis). See Supplementary Figure 1 for further details on IPC episode definition. A single IPC episode was selected per individual to reduce the influence of frequent admitters. For those with multiple episodes, one episode was selected per individual at random with equal probability to avoid outcome-dependent selection.

### Data sources

Data were extracted from five nationwide registers and linked using the pseudonymised unique personal identification number assigned to each Swedish citizen. The National Patient Register (NPR) has nationwide coverage of inpatient and specialised outpatient healthcare in Sweden^13^ and holds data on IPC since 2009.^14^ Register linkage also combined data from the Cause of Death Register (CoDR), the Total Population Register (TPR), the Longitudinal Integrated Database for Health Insurance and Labour Market Studies (LISA), and the Prescribed Drug Register (PDR).

### Clinical and general population comparison groups

We defined three comparison groups to estimate relative suicide risk in IPC patients: (i) all psychiatric inpatients, (ii) all psychiatric outpatients, and (iii) the general population. All individuals treated in psychiatric inpatient care and psychiatric outpatient care were identified via the NPR as having at least one record of voluntary or involuntary psychiatric care registered in the inpatient, and outpatient care register, respectively, within the study period (definitions in Supplementary Table 1). For individuals with more than one care episode, a single episode was selected at random with equal probability. The general population was selected from the TPR including all individuals alive and residing in Sweden within the study period. To facilitate a meaningful analysis of relative suicide risk, we randomly selected a date within the study period for each member of the general population as the start of follow-up so that the distribution of follow-up times mirrored those for the IPC patients.

### Variables

#### Outcome

Suicide was defined as any of the International Statistical Classification of Diseases and Related Health Problems, Tenth Revision (ICD-10) codes for certain (X60–X84) or uncertain (Y10–Y34) suicide as the underlying cause of death in the CoDR.

#### Clinical and socioeconomic variables

Data on legally registered sex and date of birth were available from the TPR. Clinical variables were registered in the NPR and included hospital region, admission and discharge date, primary and secondary diagnoses (Supplementary Table 2). Dispensed medications during the year prior to the discharge date were categorised according to the Anatomical Therapeutic Chemical Classification System (ATC) codes (Supplementary Table 3) as registered in the PDR. Socioeconomic variables were collated from LISA for the calendar year preceding discharge and included civil status, years since change in civil status, pension, sickness or injury benefit, unemployment benefit, and the family adjusted income. Additionally, method of suicide (Supplementary Table 4) and type of location where suicide decedents were found were recorded in the CoDR.

### Statistical analysis

For all results, we used a singular outcome definition of suicide. Missingness in data was very low or non-existent, is reported explicitly, and not imputed. All analyses were done in R (R version 4·3·1 for Windows^15^)

First, we present summary statistics on clinical and sociodemographic characteristics of IPC patients stratified by suicide. Sex-stratified suicide counts per five-year age strata are reported. Cumulative survival curves were generated for suicide stratified by primary or secondary psychiatric diagnoses and history of suicide.

Second, suicide incidence rates (IRs) were calculated per 100 000 person-years with 95% confidence intervals (CIs) for both the complete study window and for distinct follow-up times (one month, three months, one year, and five years after hospital discharge) using Poisson regression. These suicide IRs are presented for the total IPC population and across sex and age strata. Individuals were censored in case of death by other cause, migration, or end of follow-up time.

Third, crude and adjusted suicide incidence rate ratios (IRRs) were calculated for IPC patients versus (i) all psychiatric inpatients, (ii) all psychiatric outpatients, and (iii) the general population using Poisson regression with cluster-robust 95% CIs. Incidence rate ratios from Poisson models are unbiased and use of a cluster robust sandwich estimator provides accurate confidence intervals. We adjusted for the covariates sex, age at discharge, and year of follow-up start. Age was modelled both as a linear and non-linear (quadratic and cubic) effect to account for non-linearities.

Fourth, we accounted for the recurrence of psychiatric inpatient care in a secondary analysis. We included all discharge episodes from psychiatric inpatient care (voluntary or involuntary), allowing for one individual to contribute to multiple times under risk when being readmitted. Involuntary care was the exposure and voluntary care the reference. Individuals were followed from date of hospital discharge until suicide, or censoring by death from other cause, migration, readmission, or end of follow-up time. Due to extensive censoring by readmission, Cox regression was used to calculate relative risk as suicide hazard ratios (HRs) with cluster-robust 95% CIs. The proportionality assumption was checked and found to be violated. However, even with violations of the proportionality assumption, the HR can be viewed as the average HR over that specific time-period.^16^ Covariate adjustment was the same as for the IRR modelling, except for adding readmission status as an additional covariate.

### Ethics approval

The study was approved by the Swedish Ethical Review Authority on the 07.01.2021 (2020–06540). Informed consent is not required for pseudonymised register research according to Swedish regulations.

### Role of the Funding source

The funders of the study had no role in the study design, data acquisition, analysis, interpretation, or manuscript writing.

## Results

### Clinical and demographic characteristics

**Fig. 1:**
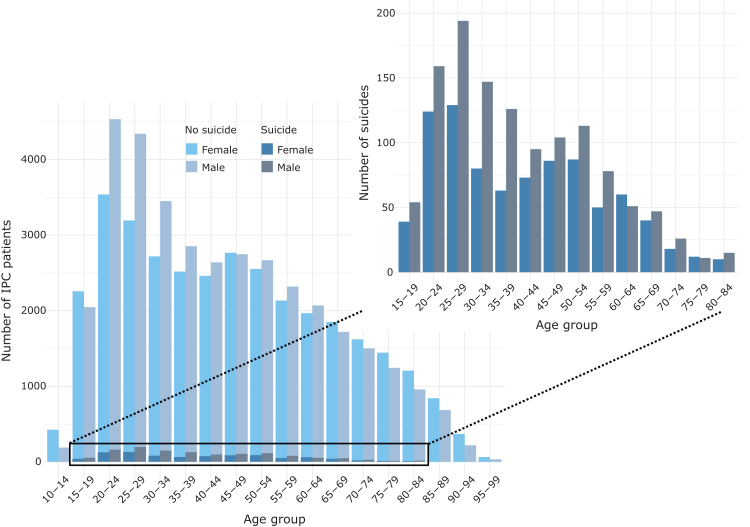
Counts of individuals who were discharged from involuntary psychiatric care, stratified by age at discharge, sex, and suicide status from 2010 through 2020. The rectangle magnifies the cases of individuals who died by suicide. Counts <5 were excluded.

From January 1st 2010 through December 31st 2020, there were 134 514 IPC episodes belonging to 72 275 unique IPC patients of which 2104 (2·9%) died by suicide. The median follow-up time was 4·4 years (IQR: 1⋅8–7⋅5). Of all suicide cases, 1623 (77·1%) were classified as certain suicides and 481 (22·9%) as uncertain suicides. On average, one suicide was recorded for every 64 discharges following IPC. Fig. 1 shows the raw count of IPC patients identified within the study window, stratified by sex, age, and suicide status. The age ranged from 6 to 101 years old. Patient sex was similarly distributed across most age strata, except for more males between 20 and 34 years. Suicide among IPC patients was more common in young and middle-aged adults, particularly among males.

Clinical characteristics of IPC patients are summarised in Table 1 (Supplementary Table 5 for statistical tests). On average, IPC patients were hospitalised for approximately four weeks before being discharged to regular housing (non-clinical). Out of the 2104 individuals who died by suicide, 38 individuals (1·8%) died during hospitalisation. Overall, IPC patients who died by suicide compared to those who did not were more likely to be diagnosed with substance use disorders (693 [32·9%] versus 16 144 [23·0%]), anxiety, dissociative, stress-related, or somatoform disorders (460 [21·9%] versus 11 526 [16·4%]), depressive disorders (451 [21·4%] versus 11 508 [16·4%]), and personality disorders (290 [13·8%] versus 4541 [6·5%]), yet less likely to be diagnosed with schizophrenia spectrum disorders (528 [25·1%] versus 22 564 [32·2%]) or organic psychotic disorders (56 [2·7%] versus 6583 [9·4%]). Also, suicide decedents were more likely to have been admitted at least once due to intentional self-harm (740 [35·2%] versus 10 211 [14·6%]) and have a history of IPC (330 [15·7%] versus 8278 [11·8%]) during the year before IPC. All dispensed medications in the year preceding IPC were more common among individuals who died by suicide, except for antipsychotics.

**Table 1:** Demographics, present clinical characteristics, and medical history of involuntary psychiatric care patients.

	Died by suicide (n = 2104)	Alive or died from other causes (n = 70 171)	Total (n = 72 275)	NA
**Demographic information**				
Male	1227 (58·3)	36 235 (51·6)	37 462 (51·8)	0 (0)
Age (mean)	40·4 (16·1)	44·9 (20·0)	44·8 (19·9)	0 (0)
Adolescents: ≤24 years	380 (18·1)	13 013 (18·5)	13 393 (18·5)	
Young adults: 25–44 years	909 (43·2)	24 171 (34·4)	25 080 (34·7)	
Middle-aged adults: 45–64 years	629 (29·9)	19 218 (27·4)	19 847 (27·5)	
Older adults: ≥65 years	186 (8·8)	13 769 (19·6)	13 955 (19·3)	
Born in Sweden	1789 (85·0)	54 291 (77·4)	56 080 (77·6)	10 (0·01)
**Clinical information**				
Inpatient stay (days)^a^	29·1 (102·5)	25·8 (62·4)	25·8 (63·9)	0 (0)
Admitted from				190 (0·25)
Ordinary housing	1575 (75·2)	56 489 (80·7)	58 064 (80·1)	
Other hospital or clinic	486 (23·2)	11 987 (17·1)	12 473 (17·3)	
Special housing	33 (1·6)	1513 (2·2)	1546 (2·1)	
Admitted to				251 (0·35)
Ordinary housing	1877 (89·5)	61 465 (87·9)	63 342 (87·9)	
Other hospital or clinic	127 (6·1)	4282 (6·1)	4409 (6·1)	
Special housing	55 (2·6)	3971 (5·7)	4026 (5·6)	
Deceased in care	38 (1·8)	211 (0·3)	249 (0·3)	
Diagnoses at discharge^b^				0 (0)
Substance use disorders	693 (32·9)	16 144 (23·0)	16 837 (23·3)	
Schizophrenia spectrum disorders	528 (25·1)	22 564 (32·2)	23 092 (32·0)	
Anxiety, dissociative, stress-related, somatoform disorders	460 (21·9)	11 526 (16·4)	11 986 (16·6)	
Depressive disorders	451 (21·4)	11 508 (16·4)	11 959 (16·5)	
Personality disorders	290 (13·8)	4541 (6·5)	4831 (6·7)	
Manic including bipolar disorders	233 (11·1)	8600 (12·3)	8833 (12·2)	
Organic psychotic disorders	56 (2·7)	6583 (9·4)	6639 (9·2)	
**Medical history**^c^				
Intentional self-harm	740 (35·2)	10 211 (14·6)	10 983 (15·2)	0 (0)
Prior IPC admission	330 (15·7)	8278 (11·8)	8608 (11·9)	0 (0)
Medications				0 (0)
Hypnotics and sedatives	1388 (70·8)	37 215 (58·3)	38 603 (58·7)	
Antidepressants	1284 (65·5)	33 493 (52·5)	34 777 (52·8)	
Antipsychotics	1183 (60·4)	40 657 (63·7)	41 840 (63·6)	
Anxiolytics	1089 (55·6)	28 169 (44·1)	29 258 (44·5)	
Antihistamines for systemic use	889 (45·4)	20 873 (32·7)	21 762 (33·1)	
Analgesics, antipyretics	594 (30·3)	15 510 (24·3)	16 104 (24·5)	
Opioids	450 (23·0)	10 195 (16·0)	10 645 (16·2)	
Antiepileptics	406 (20·7)	11 655 (18·3)	12 061 (18·3)	
Psychostimulants	231 (11·8)	4519 (7·1)	4750 (7·2)	

Data are decimal mean (SD) or integer count (%).

SD, standard deviation.

a Duration of inpatient stay including IPC.

b Primary or secondary diagnosis.

c During the year preceding IPC hospitalisation.

The socioeconomic background detailed in Table 2 (Supplementary Table 6 for statistical tests) shows that IPC patients who died by suicide were less likely to have been in a civil partnership (266 [12·7%] versus 12 742 [18·6%]) and less likely to live with a partner or child (778 [37·3%] versus 28 339 [41·3%]). Suicide decedents were also more likely to have received sickness or injury benefits prior to IPC (444 [21·3%] versus 9815 [14·3%]). Education level and the family adjusted income were similar across IPC patients who died by suicide and those who did not.

**Table 2:** Socioeconomic characteristics of involuntary psychiatric care patients.

	Died by suicide (n = 2104)	Alive or died from other causes (n = 70 171)	Total (n = 72 275)	NA
Civil status				1527 (2·11)
Single	1429 (68·5)	40 841 (59·5)	42 270 (59·7)	
Separated	350 (16·8)	12 132 (17·7)	12 482 (17·6)	
Registered partner or married	266 (12·7)	12 742 (18·6)	13 008 (18·4)	
Widowed	42 (2·0)	2946 (4·3)	2988 (4·2)	
Years spent in current civil status	27·9 (15·9)	29·1 (18·0)	29·1 (18·0)	1539 (2·13)
Living with partner or child	778 (37·3)	28 339 (41·3)	29 117 (41·2)	1527 (2·11)
Highest achieved education				11132 (15·40)
Elementary education, ≤9 years	627 (31·2)	20 551 (34·8)	21 178 (34·6)	
Secondary education, 10–12 years	938 (46·6)	26 012 (44·0)	26 950 (44·1)	
Higher education, >12 years	447 (22·2)	12 568 (21·3)	13 015 (21·3)	
Income^a^				1527 (2·11)
Q1 (lowest)	393 (18·8)	13 377 (19·5)	13 770 (19·5)	
Q2	447 (21·4)	13 583 (19·8)	14 030 (19·8)	
Q3	403 (19·3)	13 833 (20·1)	14 236 (20·1)	
Q4	372 (17·8)	13 965 (20·3)	14 337 (20·3)	
Q5	472 (22·6)	13 903 (20·2)	14 375 (20·3)	
Income support	457 (21·9)	13 143 (19·1)	13 600 (19·2)	1527 (2·11)
Number of unemployment days	21·4 (61·0)	19·6 (60·5)	19·7 (60·5)	1527 (2·11)
Unemployment benefit	99 (4·7)	2674 (3·9)	2773 (3·9)	1527 (2·11)
Sickness or injury benefit	444 (21·3)	9815 (14·3)	10 259 (14·5)	1527 (2·11)
Old age or occupation pension	255 (12·2)	15 318 (22·3)	15 573 (22·0)	1527 (2·11)

Data are decimal mean (SD) or integer count (%).

SD, standard deviation.

a Annual income adjusted for family composition and binned by quintiles.

### Absolute suicide risk

**Fig. 2:**
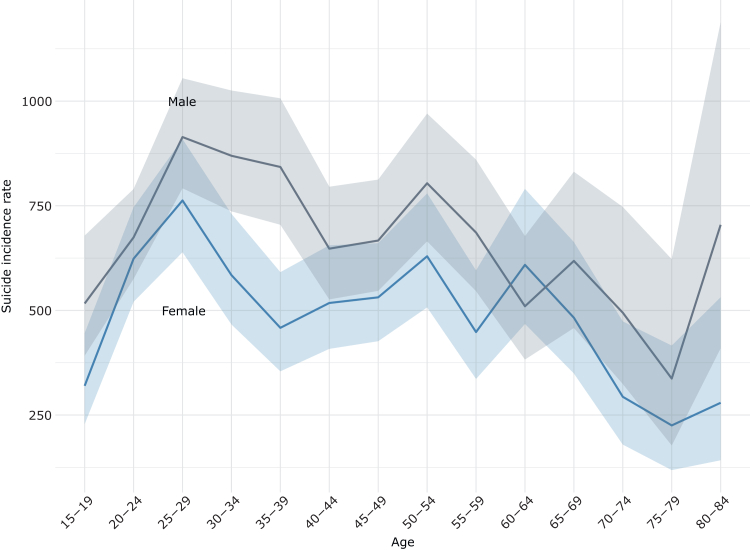
Suicide risk by sex and five-year age strata. Risk is given as incidence rate per 100 000 person-years. Male (n = 36 235) and female (n = 33 048) individuals discharged from involuntary psychiatric care from 2010 through 2020. Median follow-up time = 4·4 years (IQR: 1⋅8–7⋅5).

The suicide incidence rate following IPC over the complete follow-up period was 631 [95% CI: 605, 659] suicides per 100 000 person-years (median follow-up time = 4·4 years [IQR: 1⋅8–7⋅5]). The highest rate was the closest to discharge, one month after discharge (2941 [2537, 3407]), and decreased thereafter, such that during three months after discharge the rate was 2086 [1881, 2312], one-year 1321 [1237, 1413], and five years after discharge it was 738 [705, 773]. Suicide IRs were overall higher in males (714 [674, 755]) compared to females (530 [495, 566]). Fig. 2 depicts the suicide rate stratified by sex and five-year age strata. Many of the observed sex differences for specific age strata are however uncertain with overlapping confidence intervals. The suicide IR was highest in males of age 25–29 years (914 [792, 1055]) and lowest in females of age 75–79 years (225 [118, 416]) compared to all other strata.

**Fig. 3:**
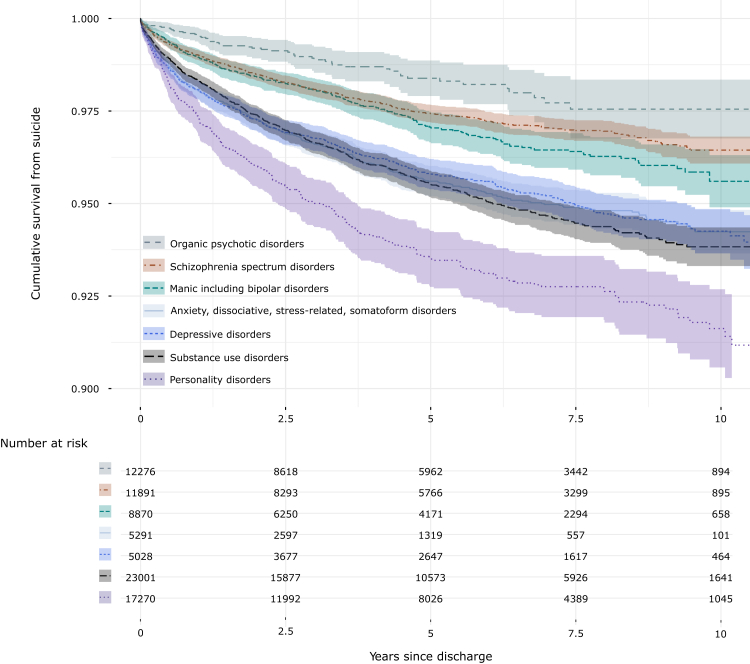
Cumulative survival from suicide after hospital discharge after involuntary psychiatric care, stratified by psychiatric diagnostic categories. Individuals discharged from IPC with a psychiatric diagnosis (n = 69 288) from 2010 through 2020. Median follow-up time = 4·4 years (IQR: 1⋅8–7⋅5).

The cumulative survival from suicide after hospital discharge in IPC patients is shown in Fig. 3. Patients diagnosed with a personality disorder had the highest suicide risk, whereas individuals with an organic psychotic disorder or schizophrenia spectrum disorder had the lowest suicide risk. In Supplementary Figure 2, the cumulative survival from suicide is shown stratified by history of IPC. Individuals with no previous admission to IPC one year before discharge had the lowest suicide risk. With each additional IPC episode, the suicide risk increased in a dose–response manner, however with overlapping confidence intervals for individuals with prior IPC episodes, likely due to limitations regarding statistical power.

### Relative suicide risk

**Fig. 4:**
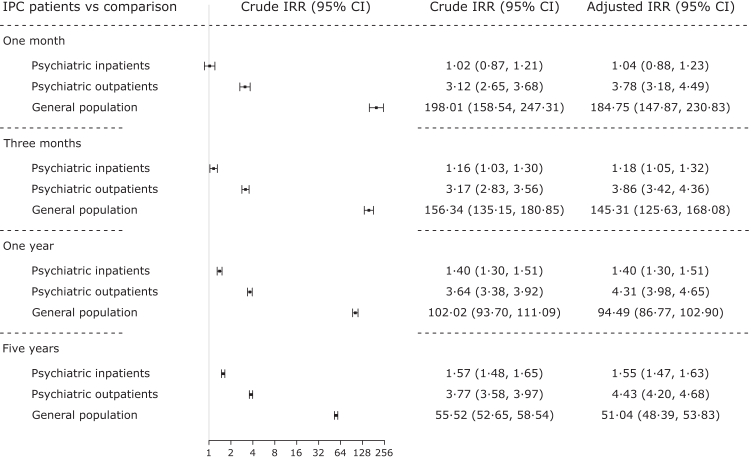
Suicide incidence rate in involuntary psychiatric care patients compared to different comparison groups. Suicide incidence rate ratios (IRRs) are given for IPC patients (n = 72 275) versus all psychiatric inpatients (n = 274 246), all psychiatric outpatients (n = 956 804), and the general population (n = 10 981 349) for different follow-up times. Adjusted for sex, age, and year of follow-up start.

Suicide incidence rate ratios (IRRs [95% CIs]) are presented in Fig. 4 for IPC patients versus (i) all psychiatric inpatients (n = 274 246), (ii) all psychiatric outpatients (n = 956 804), and (iii) the general population (n = 10 981 349). Compared to psychiatric inpatients, IPC patients had the highest relative suicide rate over all four follow-up times (IRR_5years_ = 1·57 [1·48, 1·65]), except for the period of one month post discharge when the rate was similar (IRR_1month_ = 1·02 [0·87, 1·21]). Thereafter, the relative rate increased for IPC patients compared to psychiatric inpatients with longer follow-up time. Compared to psychiatric outpatients, the relative rate was significantly elevated for IPC patients across all follow-up times and gradually increased (IRR_1month_ = 3·12 [2·65, 3·68], IRR_5years_ = 3·77 [3·58, 3·97]). Compared to the general population, the suicide rate was markedly higher in IPC patients across all follow-up times. Within the first month after discharge, the suicide rate was 198·01 [158·54, 247·32] times higher in IPC patients compared to the general population. The rate ratio decreased with longer follow-up times (IRR_5years_ = 55·52 [52·65, 58·54]). The aforementioned pattern of results remained consistent after adjusting for sex, age at discharge, and the year of follow-up start (Fig. 4), as well as when accounting for readmission (Supplementary Figure 3). When stratifying by sex, higher relative risk was observed for females across all follow-up times and comparison groups (Supplementary Figure 4).

### Suicide method, geographical variance, and seasonal trends

Regarding the method of suicide (Table 3), poisoning (794 [37·7%]) was most common, followed by hanging, suffocation, or strangulation (702 [33·4%]). Poisoning was more common in females than in males (400 [45·6%] versus 394 [32·1%]), while hanging, suffocation, or strangulation was more common in males (437 [35·6%] versus 265 [30·2%]). Almost half of all cases died in a private home (1096 [52·1%]), a fifth died in a hospital, nursing home, or sheltered accommodation (407 [19·3%]), and a fourth died at an unspecified location (557 [26·5%]). Regarding the geographical distribution of suicide rates, stratified by where IPC was given, considerable variation was observed between regions (Supplementary Figure 5). With respect to seasonal patterns, suicide cases were equally distributed over months, days, and weekdays with no clear differences emerging across calendar periods (Supplementary Figure 6).

**Table 3:** Method of suicide among involuntary psychiatric care patients stratified by sex.

	Female (n = 877)	Male (n = 1227)	Total (n = 2104)
Poisoning	400 (45·6)	394 (32·1)	794 (37·7)
Hanging, suffocation or strangulation	265 (30·2)	437 (35·6)	702 (33·4)
Jumping or lying before moving object	68 (7·8)	119 (9·7)	187 (8·9)
Drowning	53 (6·0)	52 (4·2)	105 (5·0)
Jumping from a height	39 (4·4)	82 (6·7)	121 (5·8)
Cutting or piercing	21 (2·4)	42 (3·4)	63 (3·0)
Smoke, fire, flames, steam, hot vapours, or hot objects	16 (1·8)	22 (1·8)	38 (1·8)
Gassing	6 (0·7)	23 (1·9)	29 (1·4)
Other means	6 (0·7)	14 (1·1)	20 (1·0)
Crashing of motor vehicle	··	17 (1·4)	··
Firearm or explosive	··	25 (2·0)	··

Data are count (%). Counts <5 are not presented and sums from which one can deduce such counts are excluded (··).

## Discussion

This study provides a first comprehensive and nationwide description of suicide among patients discharged after involuntary psychiatric care. Suicide rates following hospital discharge after IPC were high, with varying elevation based on sex and diagnostic categories. Overall, the risk was highest in the IPC population compared to psychiatric and community comparison groups.

Male IPC patients had on average a 35% higher risk of suicide after discharge compared to female IPC patients. This sex difference was more pronounced among younger adults. The observed trend is in line with the literature on the general population.^1^ However, the magnitude of the difference in suicide risk in IPC patients (male:female ratio = 1·35) was smaller than in the general population (male:female ratio = 2·30).^17^ This attenuation may be related to a more similar illness severity of IPC patients regardless of sex and age. Across all five-year age bands and for both sexes, the suicide rate was the highest among 25 to 29 year-olds. The same pattern was also observed in the absolute suicide numbers. These findings highlight young adulthood as a high-risk period in life for suicide following IPC discharge and warrant further research into suicide risk mitigation among young IPC patients. In addition, markers for social isolation and loneliness, such as being single, living alone, and receiving sickness benefits, were associated with an increased suicide risk. Social disparity is common among IPC patients, especially for those that later die by suicide. This seems to suggest further investigation by policy-makers into facilitating family and social support services and strengthening the community integration of individuals who experienced IPC.^18^ Psychotic conditions, including schizophrenia, were the most common diagnoses among IPC patients at hospital discharge. However, the risk of suicide was the lowest in this group compared to all other diagnostic subgroups. Instead, the highest risk was seen in those with a primary or secondary personality disorder or substance use disorder diagnosis. This warrants further investigation into the effect of specific psychiatric conditions among patients treated in IPC and might be grounds for targeted efforts on risk mitigation and follow-up post discharge tailored to fit these especially difficult-to-treat psychiatric patients. Suicide decedents were also more likely to have a history of self-harm and IPC in the year prior to discharge. The presence of a mental disorder and a history of self-harm is in line with well-known risk indicators for suicide.^1^^,^^7^^,^^19^ Besides the heterogeneity of different psychiatric disorders, also the recurrence of self-harm and IPC episodes suggest distinct psychopathological profiles and particular needs among those at higher risk for suicide. These findings should be useful for future risk stratification and could further facilitate tailored intervention for specific subgroups of IPC patients.

Suicide risk was highest during the first month following discharge from hospital. This could be explained by selection and regression to the mean, meaning in this context that a life threatening, high suicide risk can often be the reason for referral to IPC which will then at repeated measurement be lower on average.^2^ The observed exponential decrease in suicide risk over time might also be attributed to treatment leading to stabilisation of symptoms, enhanced coping strategies, and natural recovery over time. However, IPC patients remain at a high risk for suicide even five years after their IPC episode. A substantial part of their excess risk is thus long-term and suggests longer-term monitoring and follow-up after hospital discharge.

Involuntary care can be conceptualised in several ways: as a legally defined group of patients, as a proxy for illness severity, and as a coercive experience (including related interventions such as seclusion, restraint, and forced medication) that may itself influence outcomes. In most observational studies, causal inference is compromised by residual confounding. While the present observational study design precludes causal investigation, it does provide a detailed comparison of the suicide burden seen in IPC patients versus other relevant comparison populations. This underscores the value of IPC as a strong and clinically important risk indicator. While female IPC patients had a lower absolute risk of suicide compared to male IPC patients, their within-sex relative suicide risk was higher than for men. Thus, IPC itself seems be a particularly strong risk indicator for women. Regardless of comparison group and across different follow-up times, the entire IPC population had the highest suicide risk, except in comparison to psychiatric inpatients during the first month after discharge. Whether treated in IPC or not, psychiatric inpatients in general have a very high risk of suicide and typically require intensive psychiatric monitoring, treatment, and care.^3^^,^^5^^,^^6^^,^^8^^,^^20^ With longer follow-up time, the excess suicide risk in IPC patients compared to psychiatric inpatients increased – also corroborated by a secondary analysis accounting for readmission. This underscores IPC patients’ particular need for intensive psychiatric care and highlights a potential benefit of continued monitoring after intensive care is reduced.^21^^,^^22^ Compared to psychiatric outpatients, suicide risk among patients in IPC was consistently elevated, arguably reflecting substantial differences in illness severity and need of care. Compared to the general population, IPC patients showed an extremely elevated (∼200-fold) suicide risk in the month following discharge which diminished to a ∼50-fold increase for the five-year follow-up – a product of the steep absolute risk change in IPC patients following discharge and the constant low risk in the general population over time. There are several possible implications of this. One would be to investigate possibilities to target suicidality before discharge with specific suicide preventive interventions, such as Brief Cognitive Behaviour Therapy.^23^ Another is to improve follow-up care for all individuals discharged from IPC, including mental health reviews after one week, ensure safety plans are set up before discharge, and confirm that accommodation and other psychosocial needs are addressed. In addition, considerations regarding improved risk assessment could have a role. Treatments tailored to specific subgroups would likely involve more nuanced risk assessment strategies and thereby informed matching of tailored treatment modifications. Clinical trials would then be necessary for empirical testing and validation.

Regarding the method of suicide, violent suicide methods such as hanging, suffocation, or strangulation, and the use of firearms were more common in males whereas poisoning was more common in females. This pattern is in line with previous reports on suicide decedents from both psychiatric patients and the general population.^24^ The choice of more lethal methods may partially explain the elevated suicide rates in males compared to females.

### Strengths and limitations

To our knowledge, this is the first nationwide population study of suicide among IPC patients, leveraging more than a decade of detailed individual-level data on more than 70 000 unique patients subjected to more than 130 000 IPC episodes. The cohort size allowed for detailed subgroup analyses and precise risk estimation across different follow-up times. A major strength of this study is the high quality and coverage of nationwide Swedish register data, making our findings particularly generalisable to Sweden and other Nordic countries with highly similar data, healthcare systems, and sociocultural characteristics. For other European countries, our findings may also generalise well, but other nationwide studies are needed before firm conclusions can be drawn. Neither self-selection nor reporting bias are of concern for our findings.^14^^,^^25^ We were able to estimate relative suicide risk not only compared to the general population but also relative to the unselected psychiatric in- and outpatient population in Sweden. Specifically, the Swedish death register has almost complete coverage.^25^ However, there is a risk for outcome misclassification due to the acknowledged difficulty of identifying suicides.^26^ Restricting to confirmed suicide cases would underestimate the true suicide burden and systematically introduce bias, since certain methods such as hanging or firearms are more often classified as intentional suicide, while others, such as poisoning, are disproportionately coded as events of undetermined intent (uncertain suicides).^27^ Including both certain and uncertain suicides is consistent with the literature^19^^,^^28^^,^^29^ and further justified by the relatively high proportion of undetermined cases in Sweden (around 20%) compared with 2–20% reported across Europe.^30^ This definition could however result in an overestimation of absolute suicide risk. The potential misclassification could simultaneously reduce differences between groups and therefore instead produce underestimated relative risks. Misclassification by group is also theoretically possible but unlikely given the explicit registration routines of the different types of psychiatric care available in the national patient register from 2010 and onwards. As the study was based on register data only, it lacks information on psychosocial and care-related factors, such as the therapeutic relationship, patient insight, or care quality. Finally, due to the descriptive and observational design of the study, causal inferences cannot be drawn. Causal efforts building upon this descriptive foundation will be essential to account for the influence of confounders and isolate the risk associated with treatment status.

### Conclusion

The present study is the first comprehensive, nationwide description of suicide among IPC patients. We identified differences in suicide risk across age, sex, and diagnostic categories, and highlight high-risk periods following discharge from hospital. This information should motivate a more detailed analysis on the existence of different risk subgroups within IPC which could ultimately help clinicians and patients. Suicide risk among IPC patients was elevated compared with other psychiatric patients and the general population. This excess risk warrants further study as it could inform both clinicians and policy makers regarding care of patients discharged after IPC. The study addresses these critical knowledge gaps and can be used as reference for future clinical research, aiming to improve risk assessment, monitoring, and care for the scarcely studied IPC population.

## Contributors

LG, SF, PL, CR and JW conceived the research question. PL, RK-H, ZC, HL, IB, BO, CR, and JW contributed to the resources, data curation, and project administration. LG, FJ, RP, PL, RK-H, and JW contributed to the design and methods of the study. LG did the formal analysis and visualisations with support from FJ, RK-H, and JW. All authors had access to the data; LG accessed it and verified it with JW and FJ. LG and JW wrote the first draft of the manuscript. All authors reviewed, revised, and approved the manuscript. JW provided overall supervision. All authors are responsible for the decision to submit for publication.

## Data sharing statement

The Swedish population register data used in the study are available from Statistics Sweden and the Swedish National Board of Health and Welfare. According to country specific regulations and laws, the authors are not allowed to share data with third parties. Researchers interested in replicating the here presented work can apply for individual-level data from the individual Swedish population registers.

## Declaration of interest

DMC receives royalties for contributing articles to UpToDate, Inc, receives occasional honoraria for lectures, and is part owner of Scandinavian E-Health, AB, all outside the submitted work. LFC receives royalties for contributing articles to UpToDate, Wolters Kluwer Health and for editorial work from Elsevier, outside the submitted work. HL reports receiving grants from TAKEDA and Shire Pharmaceuticals; personal fees from and serving as a speaker for Medice, Shire/Takeda Pharmaceuticals and Evolan Pharma AB; all outside the submitted work. HL is editor-in-chief of JCPP Advances, unrelated to the submitted work. CR received book royalties from Studentlitteratur, Natur och Kultur and Albert Bonniers Förlag and various speaker fees, all outside the current work. SF is an expert member of the UK government Independent Advisory Panel on Deaths in Custody.
