# Supplementary material for: Suicide after involuntary psychiatric care: a nationwide cohort study in Sweden
Source: Lancet Reg Health Eur. 2025 Nov 4;60:101504. doi: 10.1016/j.lanepe.2025.101504 (PMC12637078; doi:10.1016/j.lanepe.2025.101504)
Supplement: Supplement [file mmc1.docx]

**Supplement**

For: Suicide following involuntary psychiatric care: a nationwide cohort study in Sweden

By: Leoni Grossmann, Fred Johansson, Seena Fazel, Ralf Kuja-Halkola, Björn Bråstad, David Mataix-Cols, Lorena Fernández de la Cruz, Bo Runeson, Paul Lichtenstein, Zheng Chang, Henrik Larsson, Isabell Brikell, Brian D’Onofrio, Ronnie Pingel, Christian Rück, and John Wallert.

**List of Tables**

[Supplementary Table 1: Medical field of activity codes 2](#_Toc196490356)

[Supplementary Table 2: ICD 10 codes for psychiatric diagnoses 2](#_Toc196490357)

[Supplementary Table 3: ATC codes 2](#_Toc196490358)

[Supplementary Table 4: ICD-10 codes for classifying suicide methods 2](#_Toc196490359)

[Supplementary Table 5: Demographics, present clinical characteristics, and medical history of IPC patients. 3](#_Toc196490360)

[Supplementary Table 6: Socioeconomic characteristics of IPC patients. 4](#_Toc196490361)

**List of Figures**

[Supplementary Figure 1: IPC episode definition 5](#_Toc207663085)

[Supplementary Figure 2: Cumulative survival from suicide stratified by IPC history 6](#_Toc207663086)

[Supplementary Figure 3: Adjusted relative suicide risk in involuntary versus voluntary psychiatric inpatients 7](#_Toc207663087)

[Supplementary Figure 4: Sex stratified relative suicide risk in IPC patients 8](#_Toc207663088)

[Supplementary Figure 5: Suicide risk stratified by hospital region 9](#_Toc207663089)

[Supplementary Figure 6: Daily suicide counts after IPC hospital discharge 10](#_Toc207663090)

**Supplementary Table 1: Medical field of activity codes**

| General psychiatric care, adults | 901 |
| --- | --- |
| Psychiatric nursing home care | 906 |
| Geriatric psychiatric care | 928 |
| Child and adolescent psychiatric care | 931 |
| Forensic psychiatric regional care | 943 |
| Specialized psychiatric care | 944 |
| Alcohol treatment care | 945 |
| Psychotherapeutic services | 948 |
| Social work services | 950 |
| Psychological services | 951 |
| Family care | 952 |
| Care for substance abuse | 953 |
| Care for drug addiction | 954 |
| Somatic care at psychiatric hospital | 955 |
| Forensic psychiatric evaluation services | 956 |
| Psychiatric rehabilitation | 957 |
| Care under the Communicable Diseases Act | 971 |
| Care for adults with intellectual disabilities | 991 |
| Care for children with intellectual disabilities | 993 |

**Supplementary Table 2: ICD 10 codes for psychiatric diagnoses**

| Organic psychotic disorders | F00-F09 |
| --- | --- |
| Substance use disorders | F10, T510, F11, F12, F13, F14, F15, F16, F18, F19 |
| Schizophrenia spectrum disorders | F20, F21, F22, F23, F24, F25, F28, F29 |
| Manic including bipolar disorder | F250, F30, F300, F31, F34 |
| Depressive disorder | F32, F33, F34, F38, F39 |
| Anxiety, dissociative, stress-related, somatoform disorders | F4, F400, F401, F402, F410, F411, F42, F428A, F43, F430, F431, F432, F438, F439, F438A, F452, F452A |
| Personality disorders | F60-F69 |

**Supplementary Table 3: ATC codes**

| Opioids | N02A |
| --- | --- |
| Other analgesics and antipyretics | N02B |
| Antimigraine preparations | N02C |
| Antiepileptics | N03A |
| Antipsychotics | N05A |
| Anxiolytics | N05B |
| Hypnotics and sedatives | N05C |
| Antidepressants | N06A |
| Psychostimulants, agents used for ADHD, and nootropics | N06B |
| Psycholeptics and psychoanaleptics in combination | N06C |
| Anti-dementia drugs | N06D |
| Parasympathomimetics | N07A |

**Supplementary Table 4: ICD-10 codes for classifying suicide methods**

| Poisoning | X60–X65, Y10–Y15 |
| --- | --- |
| Gassing | X66–X69, Y16–Y19 |
| Hanging, strangulation, suffocation | X70, Y20 |
| Drowning | X71, Y21 |
| Firearm or explosive | X72–X75, Y22–Y25 |
| Smoke, fire, and flames or steam, hot vapours, and hot objects | X76–X77, Y26–Y27 |
| Cutting or piercing | X78–79, Y28–Y29 |
| Jumping from a height | X80, Y30 |
| Jumping or lying before moving object | X81, Y31 |
| Crashing of motor vehicle | X82, Y3 |
| Other means | X83–X84, Y33–Y34 |

**Supplementary Table 5: Demographics, present clinical characteristics, and medical history of involuntary psychiatric care patients**

|  | Died by suicide | Alive or died from other causes | p |
| --- | --- | --- | --- |
| N | 2104 | 70 171 | 72 275 |
| **Demographic information** |  |  |  |
| Male | 1227 (58·3) | 36 235 (51·6) | <0·001 |
| Age | 40·4 (16·1) | 44·9 (20·0) | <0·001 |
| Born in Sweden | 1789 (85·0) | 54 291 (77·4) | <0·001 |
| **Clinical information** |  |  |  |
| Inpatient stay (days)* | 29·1 (102·5) | 25·8 (62·4) | 0·019 |
| Admitted from |  |  | <0·001 |
| Ordinary housing | 1575 (75·2) | 56 489 (80·7) |  |
| Other hospital or clinic | 486 (23·2) | 11 987 (17·1) |  |
| Special housing | 33 (1·6) | 1513 (2·2) |  |
| Admitted to |  |  | <0·001 |
| Ordinary housing | 1877 (89·5) | 61 465 (87·9) |  |
| Other hospital or clinic | 127 (6·1) | 4282 (6·1) |  |
| Special housing | 55 (2·6) | 3971 (5·7) |  |
| Deceased in care | 38 (1·8) | 211 (0·3) |  |
| Diagnoses at discharge** |  |  |  |
| Substance use disorders | 693 (32·9) | 16 144 (23·0) | <0·001 |
| Schizophrenia spectrum disorders | 528 (25·1) | 22 564 (32·2) | <0·001 |
| Anxiety, dissociative, stress-related, somatoform disorders | 460 (21·9) | 11 526 (16·4) | <0·001 |
| Depressive disorders | 451 (21·4) | 11 508 (16·4) | <0·001 |
| Personality disorder | 290 (13·8) | 4541 (6·5) | <0·001 |
| Manic including bipolar disorders | 233 (11·1) | 8600 (12·3) | 0·110 |
| Organic psychotic disorders | 56 (2·7) | 6583 (9·4) | <0·001 |
| **Medical history***** |  |  |  |
| Intentional self-harm | 740 (35·2) | 10 211 (14·6) | <0·001 |
| Other IPC admission | 330 (15·7) | 8278 (11·8) | <0·001 |
| Medications |  |  |  |
| Hypnotics and sedatives | 1388 (70·8) | 37 215 (58·3) | <0·001 |
| Antidepressants | 1284 (65·5) | 33 493 (52·5) | <0·001 |
| Antipsychotics | 1183 (60·4) | 40 657 (63·7) | 0·003 |
| Anxiolytics | 1089 (55·6) | 28 169 (44·1) | <0·001 |
| Antihistamines for systemic use | 889 (45·4) | 20 873 (32·7) | <0·001 |
| Analgesics, antipyretics | 594 (30·3) | 15 510 (24·3) | <0·001 |
| Opioids | 450 (23·0) | 10 195 (16·0) | <0·001 |
| Antiepileptics | 406 (20·7) | 11 655 (18·3) | 0·006 |
| Psychostimulants | 231 (11·8) | 4519 (7·1) | <0·001 |
| Data are decimal mean (SD) or integer count (%). IPC: Involuntary Psychiatric Care, SD: standard deviation. *Duration of inpatient stay including IPC. **Primary or secondary diagnosis. ***During the year preceding IPC hospitalisation. | | | |

**Supplementary Table 6: Socioeconomic characteristics of involuntary psychiatric care patients**

|  | Died by suicide | Alive or died from other causes | p |
| --- | --- | --- | --- |
| N | 2104 | 70 171 |  |
| Civil status |  |  | <0·001 |
| Single | 1429 (68·5) | 40 841 (59·5) |  |
| Separated | 350 (16·8) | 12 132 (17·7) |  |
| Registered partner or married | 266 (12·7) | 12 742 (18·6) |  |
| Widowed | 42 (2·0) | 2946 (4·3) |  |
| Years spent in current civil status | 27·9 (15·9) | 29·1 (18·0) | 0·002 |
| Living with partner or child | 778 (37·3) | 28 339 (41·3) | <0·001 |
| Highest achieved education |  |  | 0·004 |
| Elementary education, ≤9 years | 627 (31·2) | 20 551 (34·8) |  |
| Secondary education, 10-12 years | 938 (46·6) | 26 012 (44·0) |  |
| Higher education, >12 years | 447 (22·2) | 12 568 (21·3) |  |
| Income* |  |  | 0·003 |
| Q1 (lowest) | 393 (18·8) | 13 377 (19·5) |  |
| Q2 | 447 (21·4) | 13 583 (19·8) |  |
| Q3 | 403 (19·3) | 13 833 (20·1) |  |
| Q4 | 372 (17·8) | 13 965 (20·3) |  |
| Q5 | 472 (22·6) | 13 903 (20·2) |  |
| Income support | 457 (21·9) | 13 143 (19·1) | 0·002 |
| Number of unemployment days | 21·4 (61·0) | 19·6 (60·5) | 0·187 |
| Unemployment benefit | 99 (4·7) | 2674 (3·9) | 0·056 |
| Sickness or injury benefit | 444 (21·3) | 9815 (14·3) | <0·001 |
| Old age or occupation pension | 255 (12·2) | 15 318 (22·3) | <0·001 |
| Data are decimal mean (SD) or integer count (%). *Annual income adjusted for family composition and binned by quintiles. IPC: Involuntary Psychiatric Care, SD: standard deviation. P = Chi2 (categorical) or t-test (continuous) | | | |

**Supplementary Figure 1: IPC episode definition.** An inpatient care episode following IPC was defined as a consecutive in-hospital stay where the patient was at any time treated under the Swedish Compulsory Psychiatric Care Act. Inpatient care episodes were combined if they were not intermitted by ≥1 day (Scenario 1 versus Scenario 2 and Scenario 3). Forensic IPC and outpatient IPC were excluded (Scenario 4).


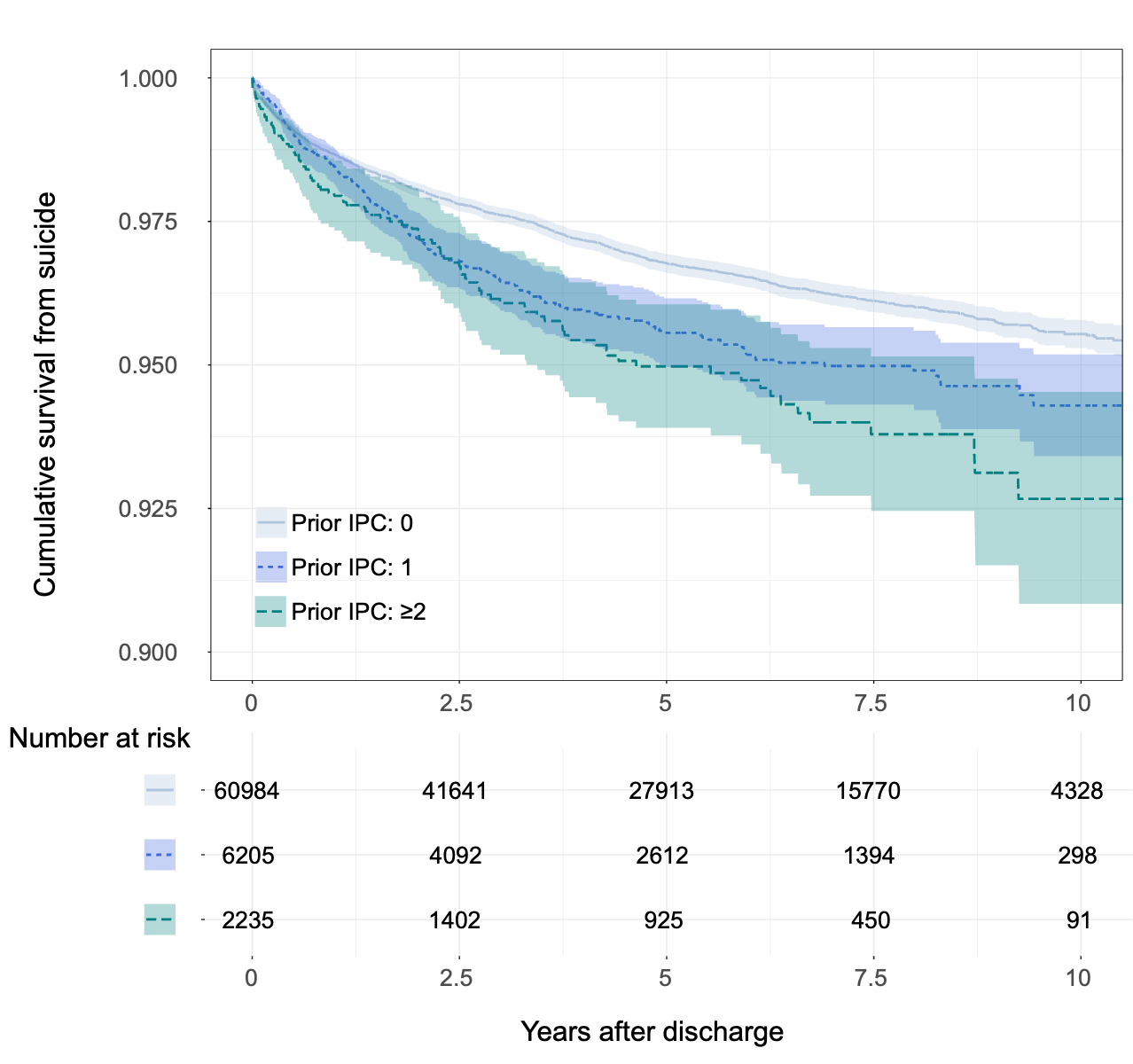


**Supplementary Figure 2: Cumulative survival from suicide stratified by IPC history**. Individuals discharged from involuntary psychiatric care (n = 69 424) from 2010 through 2020. Median follow-up time = 4·4 years.


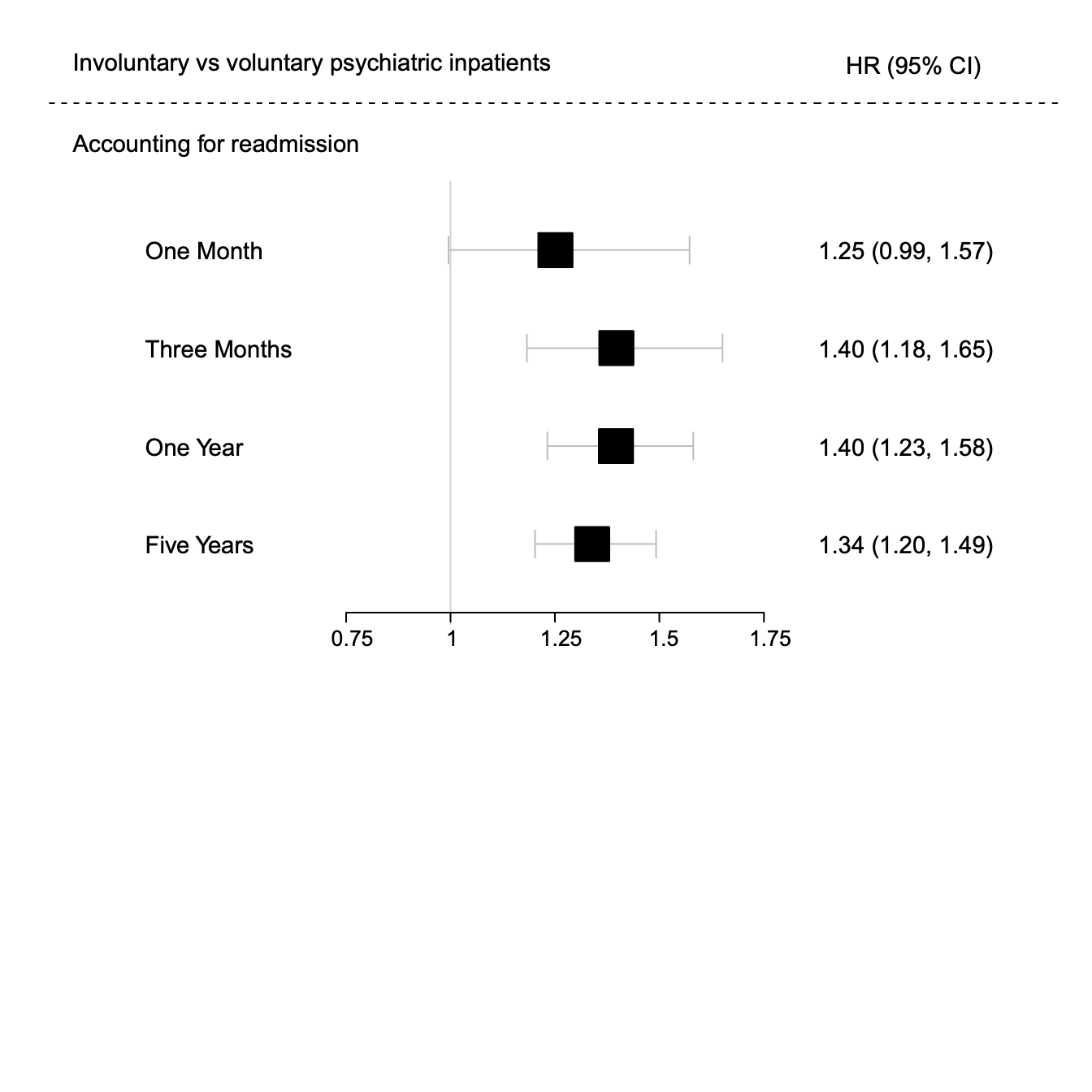


**Supplementary Figure 3: Adjusted relative suicide risk in involuntary versus voluntary psychiatric inpatients.** Relative risk for suicide is given as Hazard Ratios (HRs) across different follow-up times in psychiatric inpatients (n = 274 246), comparing involuntary versus voluntary treatment status. Adjusted for sex, age, readmission, and year of start of follow-up.


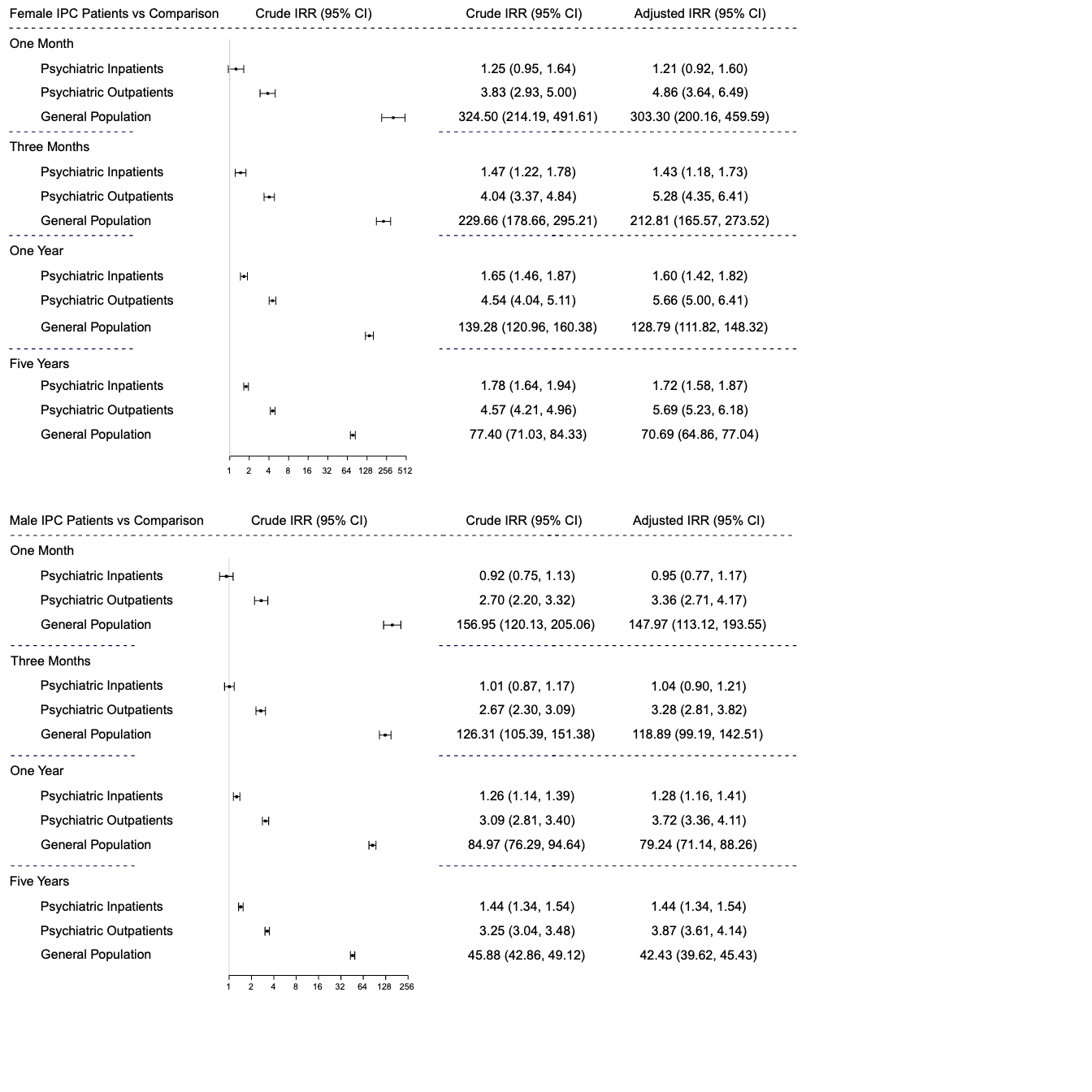


**Supplementary Figure 4: Sex stratified relative suicide risk in IPC patients compared to different populations.** Relative risk is given as IRR for female IPC patients (n = 34 738) versus female psychiatric inpatients (n = 132 577), female psychiatric outpatients (n = 496 183), and the female general population (n = 5 439 771) for different follow-up times. Relative risk is also given as IRR for male IPC patients (n = 37 346) versus male psychiatric inpatients (n = 141 669), male psychiatric outpatients (n = 460 495), and the male general population (n = 5 541 578) for different follow-up times. Adjusted for age and year of start of follow-up.

**

**Supplementary Figure 5: Suicide risk stratified by hospital region from which patients were discharged after IPC.** Risk is incidence rate per 100 000 person-years. IPC patients (n = 72 275) from 2010 through 2020.

**Supplementary Figure 6: Daily suicide counts after IPC hospital discharge aggregated over days of the week, days of the month and months of the year.** N = 2104, bars represent mean (SD). Data were aggregated over 11 years.
